# Passing Events and Topics

**Published:** 1921-03-12

**Authors:** 


### Passing Events and Topics.

### Red Cross Armistice Day Collection.

The heads of the Churches have agreed to recommend that collections should be made in aid of the work of the Council of the British Red Cross and Order of St. John next Armistice Sunday, November 13. Similar steps have been taken throughout the Empire, so that in all parts of the world worshippers will join in one common act on the same day. In America this has been already arranged. Where November 13 has, by virtue of some old custom, been reserved for a special charity, some other convenient date can be selected in the case of that particular church.

### Hospital for Nervous Diseases, Edgbaston

On Wednesday, February 23, the Lord Mayor of Birmingham (Alderman W. A. Cadbury), accompanied by the Lady Mayoress, visited the Hospital for Nervous Diseases, Edgbaston, and unveiled a tablet to the memory of the founder, Mr. Edwin Gilbart Smith, F.R.C.S. The Lady Mayoress was particularly interested in the staff quarters and the domestic arrangements. The hospital, which is the only special hospital for nervous diseases in the Midlands, has accommodation for thirty patients. Having no endowment fund, it is entirely dependent upon voluntary contributions. The necessitous poor are treated free: other patients contribute towards their maintenance during their stay in hospital according to their means.

### The League of the Roses.

On March 1 Princess Helena Victoria presented the badges and bars gained by successful workers during the past year for the League of the Roses, an organisation of the Great Northern Central Hospital. This League, which was founded in 1911, continues to show considerable vitality and progress. In its first year £300 was collected, and in 1920 the amount was £2,100, at an expense of only £138. A Highgate branch has been formed as an addition to the parent one of Islington, and it is hoped to include other localities shortly. The object of the League, which consists of women and girls, is to help towards the maintenance of the women's and children's wards of the Great Northern Central Hospital.

### The Ranyard Mission.

The Annual Sale of Work in aid of the Ranyard Mission will be held at Ranyard House, 25 Russell Square, W.C. 1, on Wednesday, March 16, from 11 a.m. to 6 p.m. The Sale will be opened by the Lady Sydenham. There are eighty Mission workers and eighty-five district nurses giving their life service for the people of London; their work was never more vital than at present, and it is essential that it shall be maintained at its highest standard. We hope the Sale of Work will be largely attended.

